# The role of dietary magnesium deficiency in inflammatory hypertension

**DOI:** 10.3389/fphys.2023.1167904

**Published:** 2023-05-24

**Authors:** Ashley Pitzer Mutchler, Linh Huynh, Ritam Patel, Tracey Lam, Daniel Bain, Sydney Jamison, Annet Kirabo, Evan C. Ray

**Affiliations:** ^1^ Vanderbilt University Department of Medicine, Division of Clinical Pharmacology, Nashville, TN, United States; ^2^ University of Pittsburgh Department of Medicine, Renal-Electrolyte Division, Pittsburgh, PA, United States; ^3^ University of Pittsburgh Department of Geology, Pittsburgh, PA, United States; ^4^ Meharry Medical College Nashville, Nashville, TN, United States

**Keywords:** magnesium, hypertension, isolevuglandins, NLRP3, dendritic cells, blood pressure, IL-1 beta, inflammasome

## Abstract

Nearly 30% of adults consume less than the estimated average daily requirement of magnesium (Mg^2+^), and commonly used medications, such as diuretics, promote Mg^2+^ deficiency. Higher serum Mg^2+^ levels, increased dietary Mg^2+^ in-take, and Mg^2+^ supplementation are each associated with lower blood pressure, suggesting that Mg^2+^-deficiency contributes to the pathogenesis of hypertension. Antigen-presenting cells, such as monocytes and dendritic cells, are well-known to be involved in the pathogenesis of hypertension. In these cells, processes implicated as necessary for increased blood pressure include activation of the NLRP3 inflammasome, IL-1β production, and oxidative modification of fatty acids such as arachidonic acid, forming isolevuglandins (IsoLGs). We hypothesized that increased blood pressure in response to dietary Mg^2+^-depletion leads to increased NLRP3, IL-1β, and IsoLG production in antigen presenting cells. We found that a Mg^2+^-depleted diet (0.01% Mg^2+^ diet) increased blood pressure in mice compared to mice fed a 0.08% Mg^2+^ diet. Mg^2+^-depleted mice did not exhibit an increase in total body fluid, as measured by quantitative magnetic resonance. Plasma IL-1β concentrations were increased (0.13 ± 0.02 pg/mL vs. 0.04 ± 0.02 pg/mL). Using flow cytometry, we observed increased NLRP3 and IL-1β expression in antigen-presenting cells from spleen, kidney, and aorta. We also observed increased IsoLG production in antigen-presenting cells from these organs. Primary culture of CD11c+ dendritic cells confirmed that low extracellular Mg^2+^ exerts a direct effect on these cells, stimulating IL-1β and IL-18 production. The present findings show that NLRP3 inflammasome activation and IsoLG-adduct formation are stimulated when dietary Mg^2+^ is depleted. Interventions and increased dietary Mg^2+^ consumption may prove beneficial in decreasing the prevalence of hypertension and cardiovascular disease.

## Background

Decades of work demonstrate that systemic inflammatory activity increases blood pressure (Rodriguez-Iturbe et al., 2017). More recent studies show that inflammation plays a key role in mediating the hypertensive effects of dietary salt (Barbaro et al., 2017; Kirabo, 2017). The NLRP3 [NOD (nucleotide-binding and oligomerization domain)-like receptor family pyrin domain containing 3] inflammasome contributes to the effect of dietary salt on blood pressure (Pitzer et al., 2022). Upon activation, NLRP3 oligomerizes and assembles with ASC (the apoptosis-associated speck-like protein containing a caspase-recruitment domain) to form a platform that induces autocatalytic cleavage of pro-caspase-1 to active caspase-1. Caspase-1, in turn, cleaves pro-IL-1β and pro-IL-18, forming IL-1β and IL-18, which are then secreted to propagate an inflammatory response (Schroder and Tschopp, 2010).

Several lines of evidence suggest the importance of the NLRP3 inflammasome in the pathophysiology of hypertension. In Dahl salt-sensitive rats, in addition to experiencing increased blood pressure, a high salt diet increases expression of NLRP3, ASC, and caspase-1 (Zhu et al., 2016). In contrast, high salt diet does not increase expression of these inflammatory mediators in Brown Norway rats, whose blood pressure is notably insensitive to dietary salt. Genetic or pharmacologic reduction of NLRP3 activity attenuates hypertension in numerous animal models of hypertension (Wang et al., 2012; Gong et al., 2016; Krishnan et al., 2016; Krishnan et al., 2019; Pitzer et al., 2020; Li et al., 2022). NLRP3 mRNA was found to be increased in kidney tissue from patients with hypertensive nephrosclerosis, suggesting a role in the pathophysiology of human hypertension (Vilaysane et al., 2010).

In addition to secreting pro-hypertensive cytokines, antigen-presenting cells (monocytes and dendritic cells) can promote hypertension through the production of isolevuglandins (IsoLGs) (Elijovich et al., 2021). These γ-ketoaldehydes are produced by peroxidation of fatty acids, including arachidonic acid, by reactive oxygen species in antigen-presenting cells. IsoLGs react with primary amines in proteins, producing essentially irreversible pyrrole adducts (Davies and May-Zhang, 2018). IsoLG-adducted peptides accumulate in antigen presenting cells and are suggested to act as neoantigens, activating CD8^+^ T cells (Bloodworth et al., 2022). CD8^+^ T cells are required to increase blood pressure in angiotensin II or mineralocorticoid/salt models of hypertension in mice (Rodriguez-Iturbe et al., 2017).

Dietary magnesium also influences blood pressure. Multiple population-based cross-sectional studies and clinical trials observed a high prevalence of magnesium deficiency with 10%–30% of individuals having serum magnesium concentrations below the standard cutoff (<0.80 mmol/L) (Lowenstein and Stanton, 1986; Kesteloot et al., 2011; Rasic-Milutinovic et al., 2012; Mejia-Rodriguez et al., 2013; Zhan et al., 2014). Dietary magnesium intake and circulating magnesium levels both correlate inversely with incidence of hypertension (Han et al., 2017; Wu et al., 2017). Magnesium supplementation reduces blood pressure in adults (Zhang et al., 2016). Dietary magnesium deficiency provokes inflammation in laboratory animals, as demonstrated by leukocytosis and increased circulating inflammatory cytokines (Weglicki et al., 1992; Malpuech-Brugère et al., 2000; Van Orden et al., 2006). In humans, magnesium supplementation reduces circulating C-reactive protein levels (Mazidi et al., 2018). We hypothesized that increased blood pressure secondary to dietary Mg^2+^ deficiency is associated with activation of the NLRP3 inflammasome and increased production of IsoLGs. We confirmed that dietary Mg^2+^ depletion in a mouse model provokes hypomagnesemia and increases blood pressure. We examined body composition of Mg^2+^-depleted mice to determine whether an increase in body fluid could contribute to this increase in blood pressure. We examined antigen-presenting cells in spleen, kidney, and aorta, discovering that Mg^2+^ depletion increased NLRP3 and IL-1β in these cells *in vivo*. Furthermore, we found that Mg^2+^ depletion increased *in vivo* formation of IsoLG-adducts. To determine whether reduced extracellular Mg^2+^ could provoke these effects directly, we examined CD11c^+^ dendritic cells in primary culture and found that reduced extracellular Mg^2+^ also increases IL-1β production. These findings suggest that systemic inflammation occurring in response to dietary Mg^2+^ insufficiency may contribute to the pathogenesis of hypertension.

## Materials and methods

### Animal care

All *in vivo* experiments were performed in mice in the *SV129* (129S2/SvPasCrl) background, obtained from Charles River Laboratories. Due to the observation that female mice are relatively resistant to inflammation-associated hypertension, only male mice were used in this study (Ji et al., 2014; Pollow et al., 2014; Veiras et al., 2021). Powdered Mg^2+^-deficient diet for mice was purchased from Envigo (Teklad TD.93106) with starting Mg^2+^ content of 0.0015%–0.003% and Na^+^ content of 0.15%. To this, Mg^2+^ oxide (Sigma) was added back to achieve final Mg^2+^ content of either 0.01%, 0.02%, or 0.08%, and in one group NaCl was added to a final Na^+^ content of 4% as noted in results section and figure legends. Powdered diets were provided to mice in cages of three to five animals in tip-proof feeding jars for powdered diets (Dyets, Inc.). Food consumption was assessed by weighing jars daily and dividing decrease in weight by the number of mice in the cage. As a positive control for NLRP3 inflammasome activation and IsoLG production in antigen-presenting cells as previously shown (Kirabo et al., 2014; Barbaro et al., 2017; Pitzer et al., 2022), mice were fed a high salt diet with NaCl added to final concentration of 4%. Blood pressures were measured non-invasively via tail cuff at baseline (while still receiving regular chow provided by the animal facility) and weekly for the duration of the treatment period, as previously described (Kirabo et al., 2011a; Kirabo et al., 2011b). Blood was collected from live mice under 2% isoflurane via left-ventricular cardiocentesis using a heparinized syringe. Blood was centrifuged in lithium heparin-containing plasma separator tubes (Becton, Dickinson and Co.). Separated plasma was removed by micropipetter, promptly frozen in liquid nitrogen, and stored at −80°C until thawing on ice for further analysis. Experiments were carried out in the Department of Laboratory Animal Research at the University of Pittsburgh or Vanderbilt University Medical Center. Animals were cared for in accordance with the Guide for the Care and Use of Laboratory Animals, U.S. Department of Health and Human Services. All Experiments were approved by these respective institutions’ Institutional Animal Care and Use Committee.

### Plasma metabolite measurement

Plasma Mg^2+^ levels were measured using a xylidyl blue assay kit (Magnesium XB Reagent Set, Pointe Scientific), as per manufacturer directions. Circulating cytokines were measured in plasma using a V-Plex ^®^ and V-Plex ^®^ Plus Mouse Proinflammatory Panel Kit (Meso Scale Diagnostics, LLC).

### Body composition analysis

Body water content was measured in non-anesthetized mice using a quantitative magnetic resonance body composition analyzer (EchoMRI) as previously described (Ray et al., 2021).

### Flow cytometry of splenic, kidney, and aortic leukocytes

Harvested spleens were placed in spleen dissociation buffer and dissociated using a 3 mL syringe plunger. Harvested kidneys were placed in kidney digestion buffer (Collagenase D (1 mg/mL; MilliporeSigma, Cat# 11088866001) and DNAse I (0.1 mg/mL; MilliporeSigma, Cat# 10104159001) and thoracic aortas with surrounding perivascular fat were placed in aorta digestion buffer (Collagenase A (1 mg/mL; MilliporeSigma, Cat# 10103578001), Collagenase B (1 mg/mL; MilliporeSigma, Cat# 11088807001), and DNAse I (0.1 mg/mL; MilliporeSigma, Cat# 10104159001) and placed in a rotating 37°C incubator for 30 min. Tissue homogenates were then passed through a 40 μm strainer and spun down at 400 × g for 10 min at 4°C. Kidney single cell suspensions underwent a Ficoll gradient centrifugation to isolate leukocytes, whereby kidney pellets were resuspended in 36% Ficoll in PBS and gently placed on top of 72% Ficoll in PBS in 15 mL tubes and centrifuged at 2,400 rpm for 15 min with decreased deceleration to prevent mixing of the density layers. Leukocytes are collected from the top density layer, diluted with PBS, and centrifuged. Single-cell suspensions were pretreated with FcR blocking reagent (Miltenyi Biotec, Cat# 130-092-575) and subsequently stained with LIVE/DEAD™ Fixable Violet Dead Cell Stain Kit (ThermoFisher) to determine cell viability. For surface staining the following fluorophore-conjugated antibodies were used (1 μg/100 μL): Brilliant Violet 510 anti-CD45 (Biolegend, Cat# 103137), PE-Cy7 anti-MerTK (Biolegend, Cat # 151522), PerCP-Cy5.5 anti-I-A/I-E (Biolegend, Cat# 107626), PE-Fluor 610 anti-CD11c (ThermoFisher, Cat# 61-01-1482), APC-Cy7 anti-CD115 (Biolegend, Cat# 135532). Cells were then fixed and permeabilized for incubation of intracellular markers. For intracellular staining, we used PE anti-IL1β (ThermoFisher, Cat# 12-71-1482), D11 ScFv antibody to detect IsoLGs which was conjugated to Alexa Fluor 488 microscale labeling Kit (ThermoFisher, Cat#A30006), and anti-NLRP3 (R&D Systems, Cat# MAB7578) which was conjugated to Alexa Fluor 647 Antibody Labeling Kit (ThermoFisher, Cat# A-20186). Samples were run on a Cytek Aurora system and analyzed using FlowJo Software (Tree Star, Inc.). Gates were set live singlets and subsequent gates were based on flow minus one (FMO) controls for each fluorophore. Results are expressed as percent positive per either dendritic cell or monocyte. Dendritic cells were identified as being CD45^+^/MerTK^−^/I-A/I-E^+^/CD11c^+^. Monocytes were identified as being CD45^+^/MerTK^−^/I-A/I-E^+^/CD11c^+^/CD115^+^. Representative flow cytometry plots were selected to best represent the gating strategy.

### CD11c^+^ dendritic cell isolation and culture

Low Mg^2+^ medium for cell culture was prepared using Gibco™ Mg^2+^-free RPMI 1640 (Life Technologies Corp.) with L-glutamine, supplemented with 1% penicillin-streptomycin, 15 mM HEPEs, 1 mM sodium pyruvate, 0.05 mM β-mercaptoethanol, and 10% fetal bovine serum. Control Mg^2+^-medium was supplemented with MgCl_2_. Final mineral concentrations were measured using inductively coupled mass-spectrometry (ICP-MS), which showed concentrations of 0.12 mM in the low Mg^2+^ solution and 0.90 mM magnesium in control Mg^2+^ solution, which is within the normal range of blood magnesium of 0.85–1.10 mM (Topf and Murray, 2003). Concentrations of calcium, potassium, and phosphorus were also measured and determined to be similar in the two media (0.72, 5.9, and 3.3 mM, respectively in the control medium; versus 0.72, 5.9, and 3.4 mM in the low Mg^2+^ medium). Dendritic cells were isolated from a single-cell suspension of splenocytes by magnetic labelling and positive selection of CD11c^+^ cells using Miltenyi Biotec Isolation Kit (Miltenyi Biotec, Cat# 130-125-835) according to the manufacturer’s protocol using LS columns. Cells were plated in 24-well plates at a density of 1 × 10^6^/mL in either low or control Mg^2+^ medium for a variable period, as described in Results section. In some experiments, cells were pre-treated for 20 min with or without YVAD (5 μg/mL, Invivogen) or MCC950 (10μM, Invivogen). IL-1β production was measured using a commercially available ELISA kit (Abcam) according to manufacturer instructions.

### Statistics

In all data sets, one mouse represents 1 *N* value. All results are presented as mean ± standard error of the mean. Normality of distribution was assessed using the Shapiro-Wilk test. Testing for outliers was performed using the ROUT method (Q = 1%). Comparisons of two groups were performed using Student’s t-tests for parametric data or Mann-Whitney test for non-parametric data. A repeated-measures analysis of variance (ANOVA) followed by a Bonferonni’s multiple comparison test were used to compare more than two groups. A comparison of more than two non-parametric groups having an N less than 6 where normality cannot be properly tested (i.e.,: Figures 3–6) a Kruskall-Wallis test with Dunn’s *post hoc* multiple comparison test were used. To analyze more than two groups compared to a control group (i.e. 0.08% Mg^2+^ diet), we used a two-way ANOVA with a Dunnett’s *post hoc* multiple comparison. For all analyses, a two-tailed *p*-values < 0.05 was used to reject the null hypothesis. Analyses were performed using Graphpad Prism 8.4.2.

## Results

**FIGURE 1:**
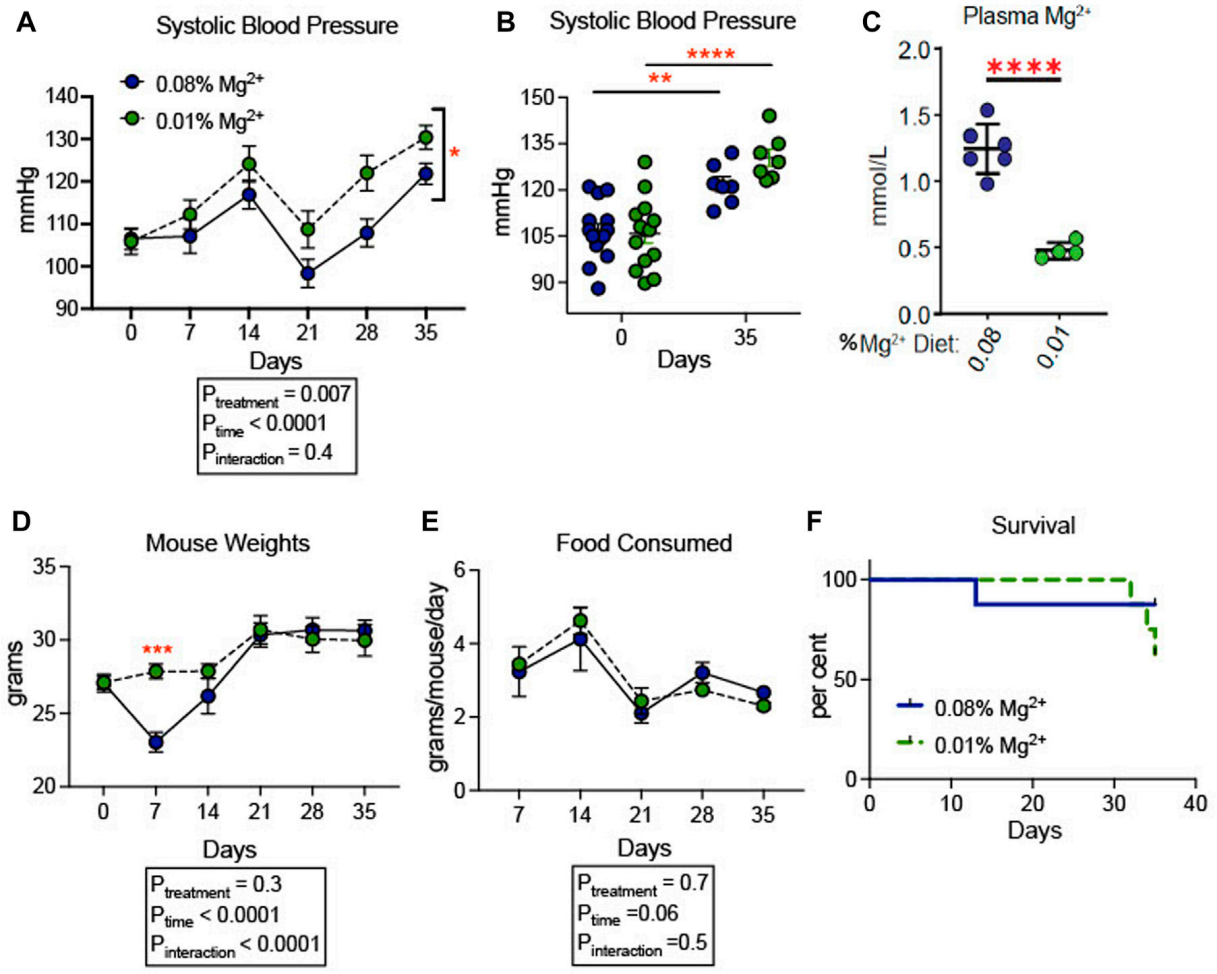
Dietary Mg^2+^ deficiency increased blood pressure in mice. **(A)** Figure showing change in systolic blood pressure for the duration of the dietary intervention (N = 7–14 per group, expressed as mean ± SEM; *p* = 0.007 for treatment effect). **(B)** Figure showing individual systolic blood pressure measurements for each mouse at day 0 (baseline) and day 35 when the experiment was terminated (N = 7–14 per group, expressed as mean ± SEM; **p = 0.002, ***p = < 0.0001). **(C)** Summarized data showing effect of dietary intervention on plasma Mg^2+^ (N = 7 for 0.08% diet, N = 5 for 0.01% diet; expressed as mean ± SEM; ****p < 0.0001 by Student’s t-test). **(D)** Mouse weights of mice fed a 0.01% Mg^2+^ or 0.08% Mg^2+^ dietary groups (*p* = 0.004 for day 7). **(E)** Food consumed per mouse per day (expressed as mean ± SEM; *p* = NS for the time-treatment interaction term). **(F)** Survival curve for the 0.08% Mg^2+^ and 0.01% Mg^2+^ dietary groups (*p* = NS by Mantel-Cox test). **(A,B,D,E)** were analyzed by two-way ANOVA with Bonferonni’s *post hoc* multiple comparisons test.

We confirmed that dietary Mg^2+^ deficiency increases blood pressure in a *SV129* background mouse model. This strain of mice was chosen because of its sensitivity to hypertensive stimuli relative to other strains of mice, such as *C57B/6J* (Zhang et al., 2020). Blood pressures increased overall in both groups of mice. Mice receiving 0.01% Mg^2+^ diet (*n* = 7), a magnesium deficient diet, developed higher blood pressures (+8.6 mmHg; Two way ANOVA *P*_*treatment*_ = 0.007) than in those receiving 0.08% Mg^2+^ diet (*n* = 7) (Figure 1A). However, both 0.01% Mg^2+^ (+15.4 mmHg; *p* = 0.002) and 0.08% Mg^2+^ (+24.6 mmHg; *p ≤* 0.001) diet groups developed significantly higher systolic blood pressure at the end of the experiment compared to baseline (Figure 1B). Mice were euthanized at the end of 5 weeks and blood Mg^2+^ was measured to confirm that mice on the 0.01% Mg^2+^ diet experienced relative hypomagnesemia (−0.8 ± 0.1 mmol/L; *p* < 0.001) compared to mice on the 0.08% Mg^2+^diet (Figure 1C). Mouse weights did not differ overall between groups, though mice in the 0.08% Mg^2+^ diet group (23.0 ± 0.67 g; *p* = 0.004), experienced a statistically significant dip in weight after the first week on the powdered diet compared to the 0.01% Mg^2+^ diet group (27.9 ± 0.5 g) (Figure 1D). Food consumption was similar between groups (Two way ANOVA, *P*_interaction_ = 0.47) (Figure 1E). Survival was similar between groups for the first 4 weeks, however in the fifth week, a series of deaths in the 0.01% Mg^2+^ diet group prompted termination of the experiment and euthanization of the mice (Figure 1F).

**FIGURE 2:**
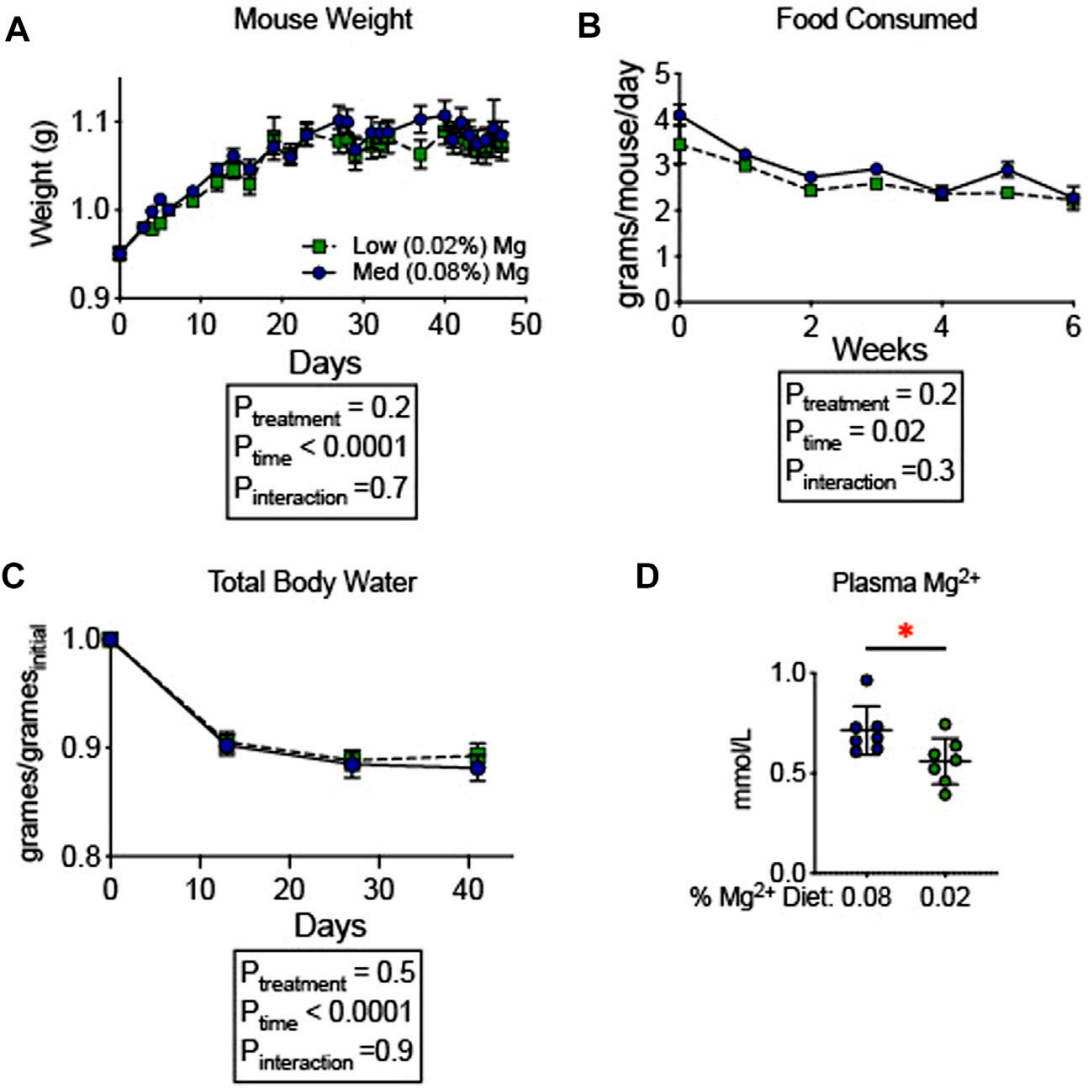
Dietary Mg^2+^ deficiency did not alter total body water. **(A)** Mouse weights on a 0.08% or 0.02% Mg^2+^ diet (N = 7 for the 0.08% Mg diet group, N = 8 for the 0.02% Mg diet group; expressed as mean ± SEM). **(B)** Total body water was measured weekly by quantitative magnetic resonance (expressed as mean ± SEM; *p* < 0.0001 for time effect; *p* = NS for the time-diet interaction effect). **(C)** Food consumed per mouse per day (expressed as mean ± SEM; *p* = NS). **(D)** Effect of the dietary intervention on plasma Mg^2+^ (expressed as mean ± SEM; *p* = 0.03 by Student’s t-test). **(A**–**C)** analyzed by two-way ANOVA with Bonferonni’s *post hoc* multiple comparisons test.

To evaluate whether the observed increase in blood pressure was associated with an increase in total body fluid, we examined the influence of dietary Mg^2+^ on body composition. A milder Mg^2+^-deficient diet (0.02% Mg^2+^, as opposed to 0.01% Mg^2+^) was employed to avoid the mortality observed in Figure 1. The 0.02% Mg^2+^ (*n* = 7) diet induced a significant reduction (−0.2 ± 0.1 mmol; *p* = 0.03) in plasma Mg^2+^ as compared to the 0.08% Mg^2+^ diet (*n* = 7), however it should be noted that the difference between 0.08% Mg^2+^ diet and the 0.02% Mg, diet was reduced compared to the difference observed in mice from Figure 1. Body composition was measured using quantitative magnetic resonance, which had previously been shown to detect increases in body water in response to mineralocorticoid activity (Morla et al., 2020) and decreases in body water in mice susceptible to body fluid volume depletion (Ray et al., 2021). Mice were placed on control (0.08%) diet for 6 days, and weights were measured. After day 6, half were transitioned to a low (0.02%) Mg diet. The remaining mice continued to receive the 0.08% diet. Weights shown are normalized to the weight the day before the transition. Overall, body weights increased to a similar degree in mice receiving the two diets (Figure 2A). Mice in both groups ate a similar quantity of food though the amount of food decreased over time for both groups (*P*_time_ = 0.02; Figure 2B). After 14 days on the powdered diet (14 days of 0.08% Mg^2+^ for one group and 7 days of 0.02% Mg^2+^ for the other group) total body water decreased in both groups by about 10% (*P*_time_ < 0.0001; Figure 2C). Over the next 4 weeks, total body water did not differ between mice on the 0.02% or the 0.08% Mg^2+^ diet (*P*_interaction_ = 0.9). Thus, we found no evidence that dietary Mg^2+^ depletion altered total body fluid.

**FIGURE 3:**
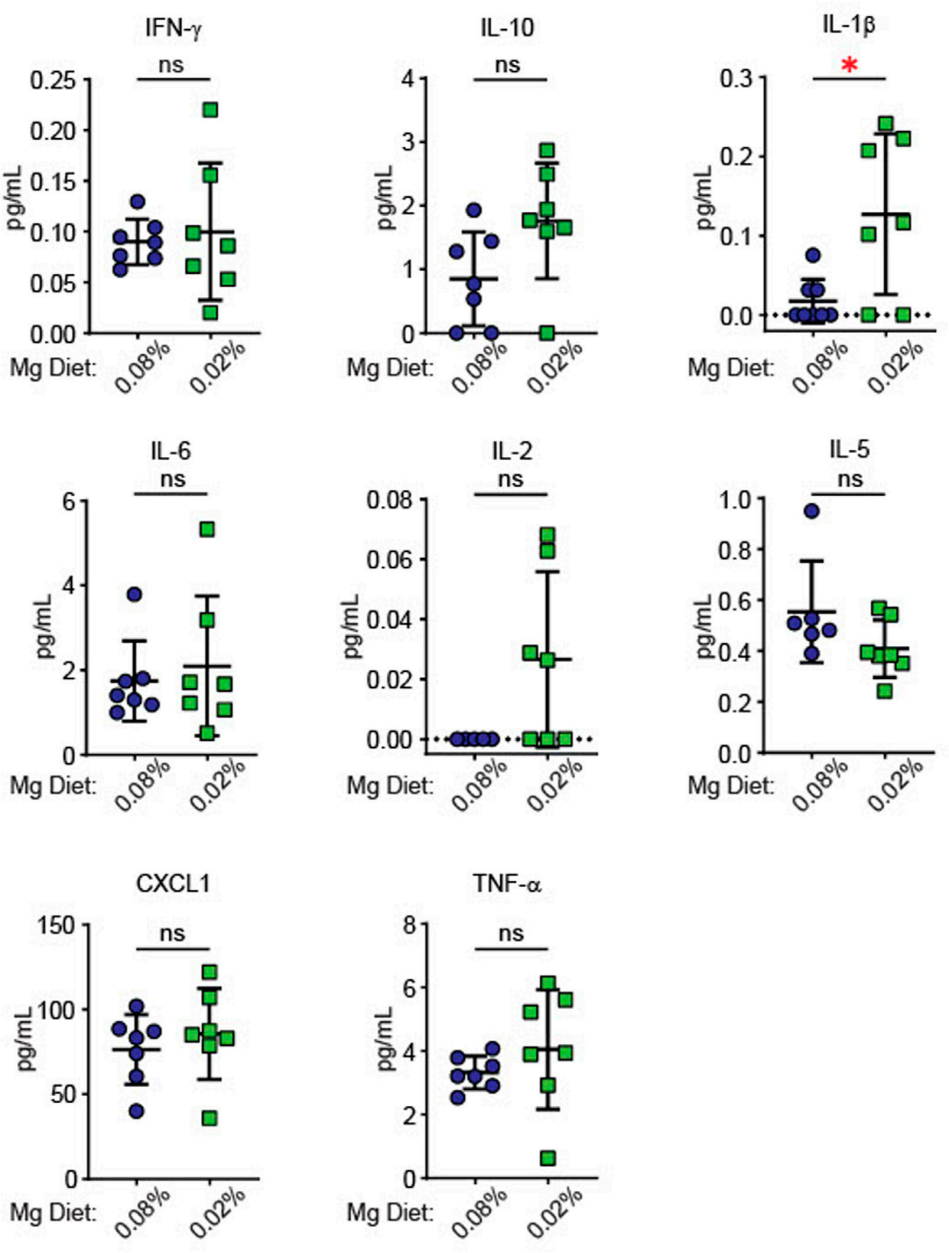
Low Mg^2+^ diet significantly increased plasma IL-1β. Figure shows levels of cytokines in the plasma from both the 0.08% Mg^2+^ and 0.01% Mg^2+^ diet fed mice of Figure 2 (N = 7 for the 0.08% Mg diet group, N = 8 for the 0.02% Mg diet group; expressed as mean ± SEM; *: *p* = 0.01 by Student’s t-test).

Since inflammatory activity increases blood pressure in models of hypertension (Drummond et al., 2011; Iulita et al., 2018; Hengel et al., 2022), we examined whether dietary Mg^2+^ deficiency induced systemic changes in inflammatory cytokines. Cytokines were measured in plasma of mice euthanized at the end of the experiment in Figure 2, after 6 weeks on either a 0.08% Mg^2+^ or 0.02% Mg^2+^ diet. We found a significant increase in IL (interleukin)-1β in mice on the 0.02% Mg^2+^ (0.02% Mg^2+^, 0.24 ± 0.1 versus 0.08% Mg^2+^, 0.08 ± 0.03 pg/mL; *p* = 0.02) diet (Figure 3). Point estimates for tumor necrosis factor alpha (TNF-α), IL-2, and IL-10 were higher but were not significantly different. Levels of IFN (interferon)-γ, IL-6, IL-5, and chemokine C-X-C motif ligand 1 (CXCL1) were not different.

**FIGURE 4:**
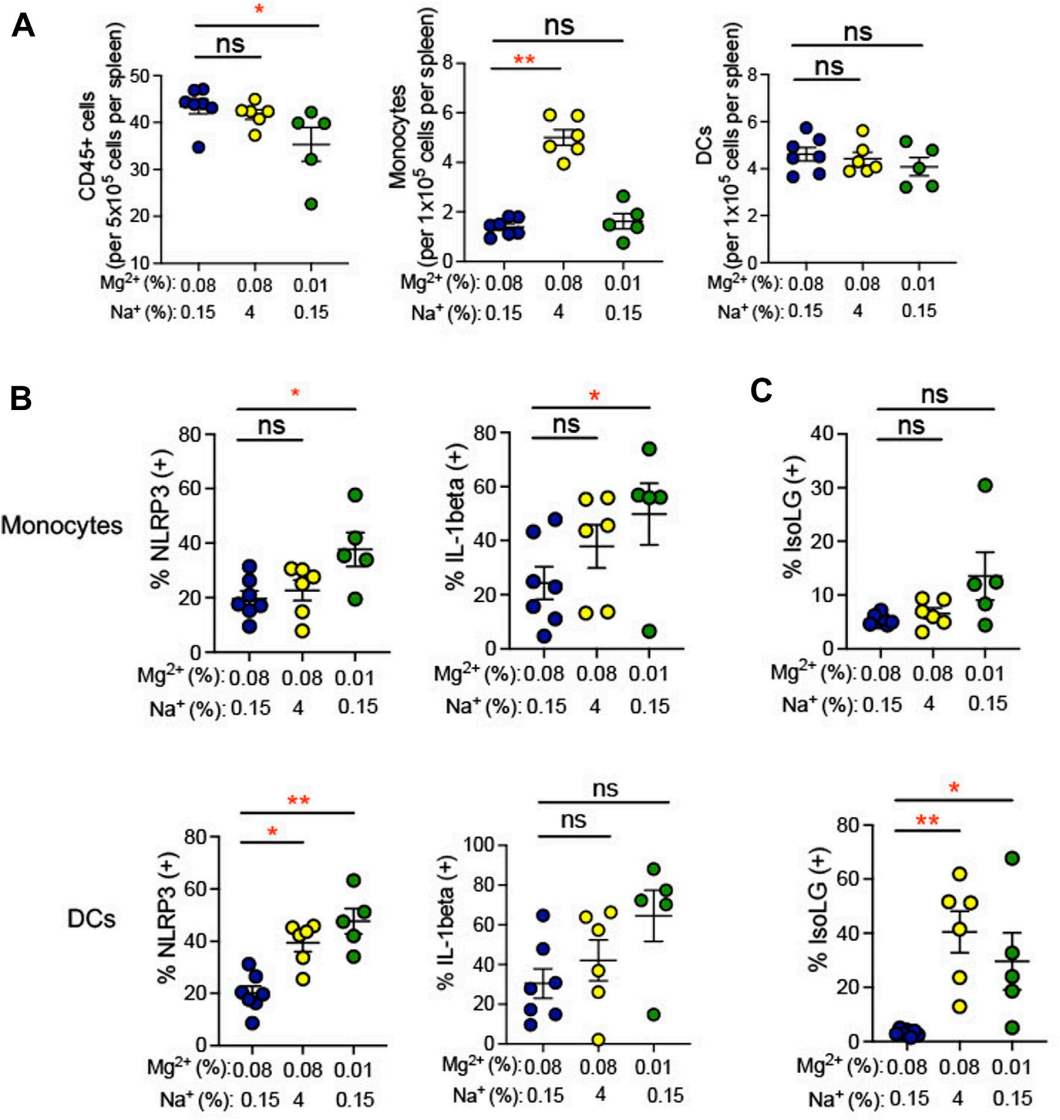
Low Mg^2+^ diet increased indicators of inflammatory activity in the spleen. **(A)** Flow cytometric analysis of total leukocytes (CD45^+^ cells), monocyte counts, and dendritic cell counts per spleen. **(B)** NLRP3 inflammasome and IL-1β cytokine positive cells are shown in splenic monocytes from animals on a standard (0.08%) Mg^2+^, standard (0.15%) Na^+^ diet, as compared to animals on a standard Mg^2+^, high (4%) salt diet or on a low (0.01%) Mg^2+^, standard (0.15%) Na^+^ diet. **(C)** Summarized data showing effect of 0.08% Mg^2+^, high salt, and 0.01% Mg^2+^diet on IsoLG positivity in splenic dendritic cells and monocytes. Data for **(A**–**C)** are expressed as mean ± SEM; *p < 0.05; **p < 0.01 by Kruskall-Wallis test with a Dunn’s *post hoc* multiple comparisons test.

Plasma cytokine levels are a relatively insensitive indicator of systemic inflammation. We therefore looked more directly at expression of IL-1β in splenic antigen-presenting cells (monocytes and dendritic cells). Single cell suspensions prepared from spleens of mice euthanized at the end of the experiment in Figure 1 and were used for flow cytometry gated to detect total leukocytes and either dendritic cells or monocytes. Gating strategy for flow cytometric analysis can be found in Supplementary Figure S1. We found that total splenic CD45^+^ leukocytes decreased in response to the 0.01% Mg^2+^ diet with no changes in either dendritic cell or monocyte numbers (Figure 4A). Additionally, we also examined expression of NLRP3, which we have previously shown to increase in association with a high salt diet. Increased NLRP3 inflammasome activity is necessary for high salt diet-induced increase in IL-1β expression and for increased blood pressure in an angiotensin II-primed mouse model of hypertension (Pitzer et al., 2022). We found that the percentages of IL-1β expressing splenic monocytes (0.01% Mg^2+^, 49.83% ± 11.3% versus 0.08% Mg^2+^, 24.4% ± 6.1%; *p* = 0.04) but not dendritic cells increased significantly (Figure 4B). The percentage of IL-1β expressing cells failed to increase in response to high salt diet. In contrast, the 0.01% Mg^2+^ diet increased percent NLRP3 positivity significantly in both splenic monocytes (0.01% Mg^2+^, 37.7% ± 6.2% versus 0.08% Mg^2+^, 19.7% ± 2.7%; *p* = 0.03) and dendritic cells (0.01% Mg^2+^, 47.6% ± 4.9% vs. 0.08% Mg^2+^, 20.1% ± 2.7%; *p* = 0.002).

Additionally, we examined isolevuglandins (IsoLGs) in splenic antigen-presenting cells. These γ-ketoaldehydes arise through oxidation of fatty acids and their phospholipid esters, in particular prostaglandin H_2_ (Salomon and Bi, 2015). In animal models of hypertension, IsoLG-protein adducts accumulate in antigen-presenting cells, and scavenging of IsoLGs attenuates the increase in blood pressure observed in these models (Kirabo et al., 2014; Barbaro et al., 2017; Pitzer et al., 2022). We observed an increase in the percentage of IsoLG positive splenic dendritic cells (4% NaCl, 5.8% ± 1.4% versus 0.08% Mg^2+^, 0.6% ± 0.5%; *p* = 0.01), but not monocytes, in mice subjected to a high salt diet (Figure 4C). Dietary Mg^2+^ deficiency with a 0.01% Mg^2+^ diet induced increases in the percentage of IsoLG^+^ dendritic cells (0.01% Mg^2+^, 8.5% ± 2.0% vs. 0.08% Mg^2+^, 0.6% ± 0.5%; *p* = 0.002) but not monocytes, suggesting that Mg^2+^ depletion is a potent stimulus of this pro-hypertensive inflammatory pathway.

**FIGURE 5:**
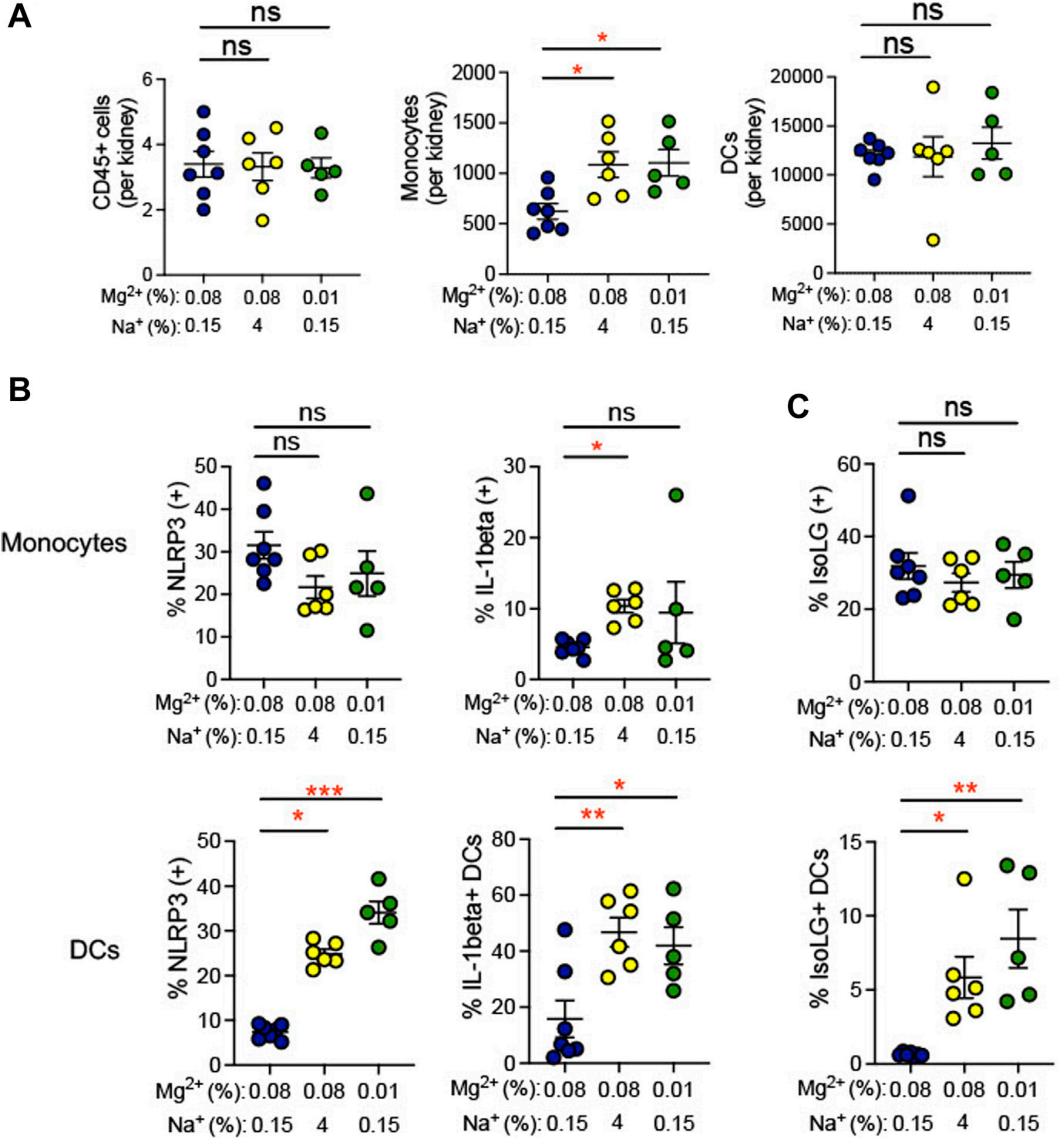
Low Mg^2+^ diet increased indicators of inflammatory activity in the kidney. Mice examined were the same as those from Figure 4. **(A)**. Flow cytometric analysis of total leukocytes (CD45^+^ cells), monocyte counts, and dendritic cell counts per spleen. Figure showing the effect of 0.08% Mg^2+^, high salt diet, and 0.01% Mg^2+^ diet on NLRP3 inflammasome or IL-1β positivity **(B)** or IsoLG-adduct formation **(C)** positivity in kidney monocytes and dendritic cells. Results from animals on high salt or 0.01% Mg^2+^ diets were compared to animals on the 0.08% Mg^2+^ diet using a Kruskall-Wallis with Dunn’s *post hoc* multiple comparisons test (expressed as mean ± SEM;*p < 0.05; **p < 0.01; ***p < 0.001).

Inflammation in the kidney has been suggested to promote hypertension (Elijovich et al., 2021). We observed an increase in infiltrating monocytes (0.01% Mg^2+^, 8.5% ± 2.0% versus 0.08% Mg^2+^, 0.6% ± 0.5%; *p* = 0.002), but not dendritic cells or total CD45^+^ leukocytes, in mice fed a 0.01% Mg^2+^ diet (+483.0 ± 131.7; *p* = 0.03) as well as the mice fed a high salt diet (+464.0 ± 131.7; *p* = 0.03) when compared with the 0.08% Mg^2+^ diet group (Figure 5A). Additionally, we examined whether dietary Mg^2+^ deficiency promotes NLRP3 inflammasome activation and IsoLG formation in the kidney. Renal monocytes did not exhibit increased NLRP3 or IL-1β positivity in either mice receiving a 0.01% Mg^2+^ diet or a high salt diet. In contrast, the percentage of dendritic cells that were positive for IL-1β was dramatically enhanced (0.01% Mg^2+^, 41.9% ± 6.6% versus 0.08% Mg^2+^, 15.9% ± 6.6%; *p* = 0.03), as was the percentage that was positive for NLRP3 (0.01% Mg^2+^, 34.06% ± 2.5% versus 0.08% Mg^2+^, 7.4% ± 0.6%; *p* = 0.0004), (Figure 5B). As with cytokine activity, monocytes did not exhibit an increase in percent positivity for IsoLGs either in the context of the 0.01% Mg^2+^ diet or the 4% Na^+^ diet. Both the 0.01% Mg^2+^ diet (+7.83 ± 2.0%; *p* = 0.002) and the high salt diet (+5.2 ± 1.4%; *p* = 0.01) increased the percent IsoLG positive kidney dendritic cells compared to the 0.08% Mg^2+^ diet (Figure 5C). These results confirm that dietary Mg^2+^ deficiency, similar to high dietary salt intake, can enhance immune activity in the kidney, a key regulator of systemic blood pressure.

**FIGURE 6:**
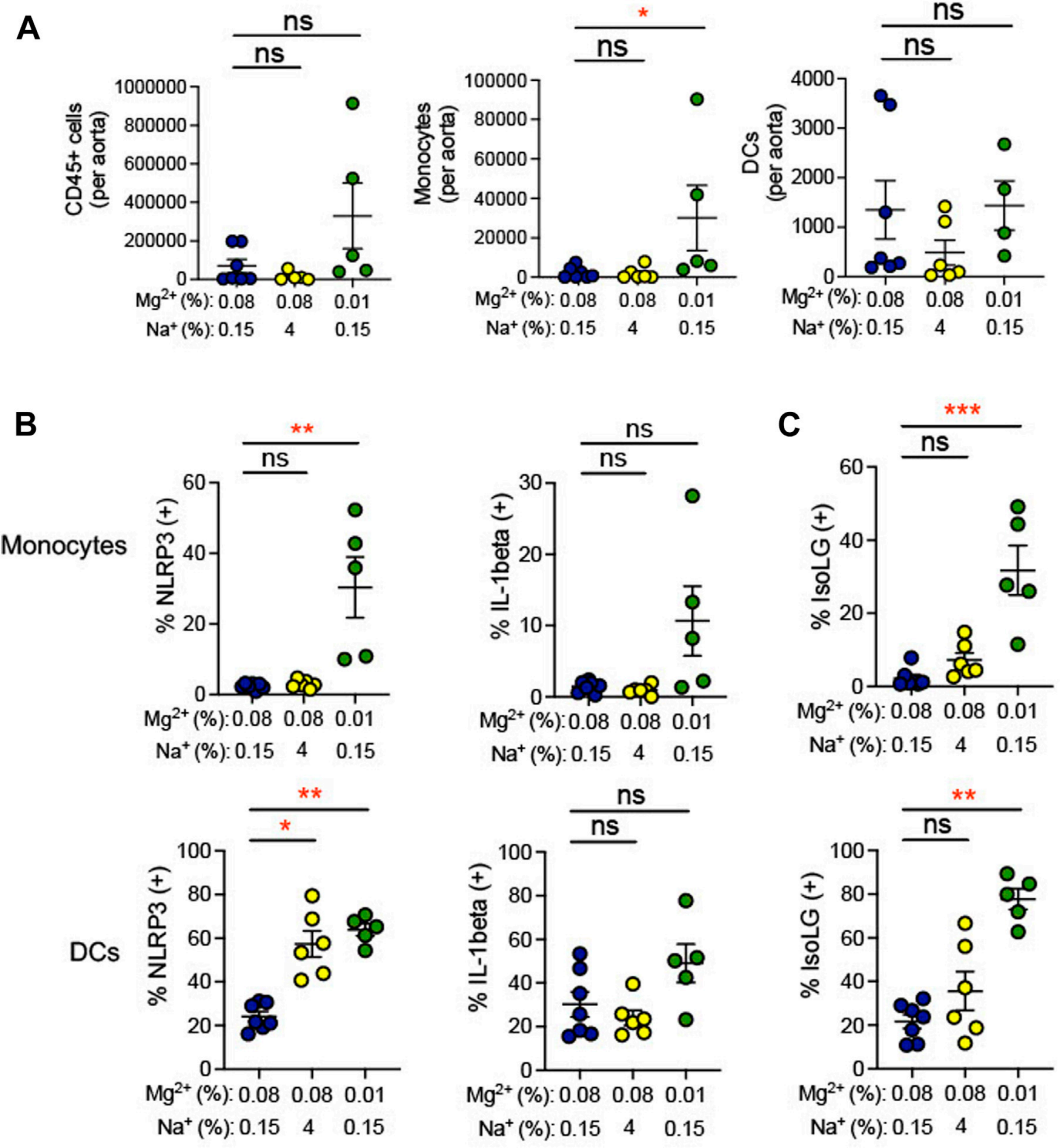
Low Mg^2+^ diet increased indicators of inflammation in the aorta. Mice examined were the same as those from Figure 4. **(A)** Flow cytometric analysis of total leukocytes (CD45^+^ cells), monocyte counts, and dendritic cell counts per spleen. Figure showing the effect of 0.08% Mg^2+^, high salt diet, and 0.01% Mg^2+^ diet on NLRP3 inflammasome of IL-1β positivity **(B)** or IsoLG-adduct formation **(C)** positivity in aortic monocytes and dendritic cells. Results from animals on high salt or 0.01%Mg^2+^ diets were compared to animals on a 0.08% Mg^2+^ diet using a Kruskall-Wallis test with a Dunn’s *post hoc* multiple comparisons test (expressed as mean ± SEM;*p < 0.05; **p < 0.01; ***p < 0.001).

During hypertension, immune cells have been shown to transmigrate into the vasculature and produce cytokines, which promote endothelial dysfunction, increasing blood pressure (Trott et al., 2014). Indeed, we found that the 0.01% Mg^2+^ diet significantly increased the number of monocytes per aorta (+27,818 ± 16,593; *p* = 0.04) but not dendritic cells nor total CD45^+^ leukocytes (Figure 6A). We therefore asked whether low Mg^2+^ diet influences inflammation and oxidative stress in aortic antigen-presenting cells. The 0.01% Mg^2+^ diet had no effect on percent IL-1β positivity in aortic dendritic cells or monocytes. However, it increased NLRP3 positivity in both monocytes (0.01% Mg^2+^, 30.4% ± 8.6% versus 0.08% Mg^2+^, 2.4% ± 0.3%; *p* = 0.004) and dendritic cells (0.01% Mg^2+^, 63.8% ± 2.8% versus 0.08% Mg^2+^, 24.2% ± 2.3%; *p* = 0.004), even though the high salt diet only increased NLRP3 percent positivity in dendritic cells (4% NaCl, 57.3% ± 6.0% versus 0.08% Mg^2+^, 24.2% ± 2.3%; *p* = 0.02), but not monocytes (Figure 6B). Additionally, the 0.01% Mg^2+^ diet dramatically increased the percentage IsoLG positive monocytes (+29.5 ± 6.8%; *p* = 0.0008) and dendritic cells (+56.0 ± 4.7%; *p* = 0.004) compared to the 0.08% Mg^2+^ diet (Figure 6C). Thus, dietary Mg^2+^ deficiency in this model system potently stimulates immune activity in the aorta.

**FIGURE 7:**
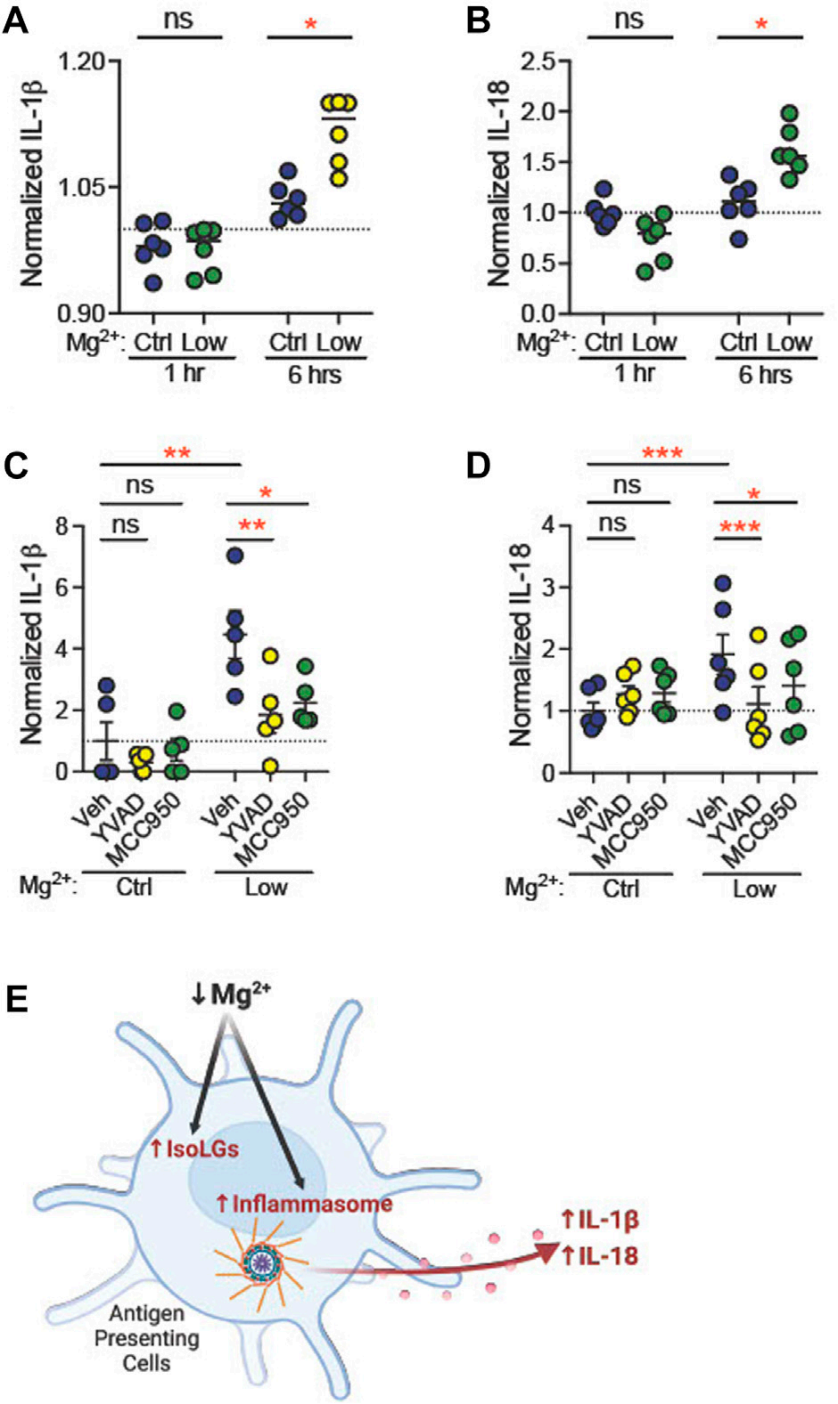
Low extracellular Mg^2+^ directly stimulates dendritic cell IL-1β production. CD11c^+^ dendritic cells isolated from a splenocyte cell suspension were subjected to control (0.92 mM) Mg^2+^ or low (0.12 mM) Mg^2+^ for 1 h or 6 h, and IL-1β **(A)** and IL-18 **(B)** was measured in cell medium. Data shown are normalized to IL-1β levels in medium from cells in control Mg^2+^ at the same time-point (N = 6, expressed as mean ± SEM). CD11c^+^ dendritic cells were cultured for 18 h in control or low Mg^2+^ medium, with either vehicle, a caspase-1 inhibitor (YVAD, 5 μg/mL), or an NLRP3 inhibitor (MCC950, 10 µM). Data shown are normalized values of ELISA for mature IL-1β **(C)** and IL-18 **(D)** production (N = 5, 6, expressed as mean ± (SEM)) **(E)** Graphical representation showing Mg^2+^ deficiency increases NLRP3 inflammasome activation and IsoLG production in antigen presenting cells. (Figure produced with Biorender.com.) Pairwise comparisons were performed using a two-way ANOVA with Tukey’s *post hoc* test for multiple comparisons (expressed as mean ± SEM;*p < 0.05; **p < 0.01***p < 0.001).

Dietary Mg deficiency could stimulate dendritic cells directly, or through systemic effects. To determine whether Mg^2+^ depletion directly stimulates NLRP3 expression and IsoLG production, we isolated CD11c^+^ dendritic cells and incubated them *in vitro* in medium containing either low Mg^2+^ (0.12 mM) or control (0.90 mM) Mg^2+^. By 6 h, both IL-1β levels (Ctl, 1.03 ± 0.01, *n* = 6; Low, 1.12 ± 0.02, *n* = 6; *p* = 0.04; Figure 7A) and IL-18 levels (Ctl, 1.1 ± 0.1, *n* = 6; Low, 1.6 ± 0.1, *n* = 6; *p* = 0.02; Figure 7B) in the medium increased significantly.

Caspase-1 mediates production of IL-1β from pro-IL-1β (Xu and Nunez, 2022). Caspase-1, in turn, is activated through the actions of active NLRP3. We asked whether inhibition of caspase-1 or NLRP3 attenuates stimulation of dendritic cell IL-1β production in low Mg^2+^ medium. CD11c^+^ dendritic cells were incubated in the presence of vehicle (dH_2_O), a caspase-1 inhibitor (5 μg/mL YVAD) or an inhibitor of NLRP3 (10 μM MCC950, also known as CP-456,773) (Perregaux et al., 2001). Eighteen hours in low Mg^2+^ solution increased IL-1β (*n* = 5, Figure 7C) concentration in cell medium (fold change, +4.5 ± 0.8; *p* = 0.002)**.** Similarly, we observed an increased release of IL-18, another NLRP3 inflammasome activation marker (*n* = 6, Figure 7D)**,** in the cell culture medium (fold change, +1.9 ± 0.3; *p* = 0.003). Both YVAD and MCC950 significantly attenuated this increase in IL-1β (YVAD, *p* = 0.006; MCC950, *p* = 0.01; Figure 7C) and IL-18 concentration (YVAD, *p* = 0.003; MCC950, *p* = 0.04**;** Figure 7D). These data suggest that NLRP3 and caspase-1 are necessary for stimulation of IL-1β and IL-18 production in low Mg^2+^ medium.

## Discussion

The present study demonstrates that moderate dietary Mg^2+^ depletion increases blood pressure in a mouse model. This increase in blood pressure is achieved with no significant difference in total body fluid. Dietary Mg^2+^ depletion also stimulated systemic inflammation, as seen by an increase in circulating IL-1β levels. Mg^2+^ depletion increased NLRP3 expression in monocytes from spleen and aorta and in dendritic cells from spleen, kidney, and aorta. Mg^2+^ depletion also induced IsoLG production in monocytes from spleen and aorta and in dendritic cells from spleen, kidney, and aorta (Figure 7E). The effects of dietary Mg^2+^-depletion on these markers of inflammation were generally comparable to, or greater than, the effects of a high salt diet. Finally, Mg^2+^-depletion in dendritic cells in culture induced IL-1β production, confirming a direct stimulatory effect.

In humans, systemic magnesium depletion is common. Content of essential minerals, including Mg^2+^, in contemporary fruits and vegetables has declined by 80% or more compared to Mg^2+^ in produce grown in the early 20th century (Workinger et al., 2018). Food Mg^2+^ is also reduced by modern processing methods (Rosanoff and Kumssa, 2020). As such, nearly a third of any given population in developed countries consume less than their estimated average daily Mg^2+^ requirement (Moshfegh et al., 2009; Costello et al., 2016). Moreover, commonly used medications further promote systemic Mg^2+^ depletion (Ray et al., 2022). Thiazide-type diuretics, one of the most commonly employed classes of clinical anti-hypertensive agents, induce urinary Mg^2+^ wasting, promoting hypomagnesemia (Kieboom et al., 2018). Hydrochlorothiazide, for example, was the 11th most commonly prescribed medication in the United States in 2020 (SP, 2022). Thus, Mg depletion is rampant.

Mg^2+^-depletion promotes hypertension. In Wistar rats given a Mg^2+^-deficient diet, blood pressure increased (Murasato et al., 1999). Dietary Mg^2+^ supplementation attenuated increases in blood pressure in both angiotensin II and high salt diet rat models of hypertension (Pere et al., 2000; Finckenberg et al., 2005). However, not every animal study has shown increases in blood pressure with Mg^2+^-depletion. An early study in Wistar rats showed decreased blood pressure (Itokawa et al., 1974). Animals in this study exhibited extracellular fluid overload, suggesting systemic illness. Another study showed no difference in blood pressure (Laurant et al., 1999). In that study, Wistar rats were 3 weeks of age, which may have influenced blood pressure.

Human studies suggest that dietary Mg^2+^ protects against increased blood pressure. Lower serum Mg^2+^ was found to be associated with increased odds of hypertension (Posadas-Sanchez et al., 2016). Dietary Mg^2+^ in-take was associated with decreased risk of hypertension in a meta-analysis of ten cohort studies (Han et al., 2017). Meta-analyses of randomized controlled trials suggest a dose-dependent reduction in blood pressure in persons given supplemental Mg^2+^ (Kass et al., 2012). This effect was found to be more significant in hypertensive individuals (Rosanoff and Plesset, 2013; Rosanoff et al., 2021). These studies provide a compelling case that systemic Mg^2+^ balance influences blood pressure in humans.

Systemic Mg^2+^ depletion could increase blood pressure through numerous mechanisms. In our study, although a low Mg^2+^ diet increased blood pressure, it did not increase total body fluid. The decreased difference in plasma Mg^2+^ level observed in mice examined for body composition, as compared to mice examined for blood pressure, represents a caveat in the interpretation of these findings. It is possible that a difference in body water may have been observed if experimental and control groups had a bigger difference in plasma Mg^2+^ or if body water had been measured at more frequent time intervals. Nevertheless, the data do not provide evidence for a difference in body water associated with hypomagnesemia.

Hypertension and kidney disease are closely linked whereby hypertension can also cause as well as contribute to kidney disease progression (Kestenbaum et al., 2008). Incidence and severity of hypertension increases as the estimated glomerular filtration rate (eGFR), a marker of kidney damage, increases (Baekken et al., 2008). NLRP3 inflammasome activation promotes inflammation, tubular injury, and fibrosis and is involved in pathogenesis of chronic kidney disease (Vilaysane et al., 2010). In a large multiethnic population-based cohort, low serum Mg^2+^ levels were associated with decreased eGFR (Ferre et al., 2019). Additional studies have shown low baseline serum Mg^2+^ levels associated with higher risk for end stage renal disease (Sakaguchi et al., 2015). In our study, mice on a low Mg^2+^ diet exhibited elevated systolic blood pressure and increased NLRP3 inflammasome activation in kidney dendritic cells, which may contribute to intrarenal inflammation associated with magnesium deficiency-induced hypertension.

Differences in arterial elasticity may contribute to the observed increase in blood pressure in mice on a low Mg^2+^ diet. Increased systemic Mg^2+^ has been shown to increase arterial elasticity (Laurant et al., 1999; Van Laecke et al., 2011). Arterial elasticity, in turn, is influenced by cardiovascular inflammation (Zanoli et al., 2020). Mg^2+^ mediates vasorelaxation through its action as a calcium channel antagonist, regulating calcium influx (Bara and Guiet-Bara, 2001). Hypomagnesemia has been associated with vascular stiffness and endothelial dysfunction, which is a predictive measure for cardiovascular mortality in patients with essential hypertension (Kisters et al., 2006). Given the absence of a change in volume status, the modest increase in blood pressure observed in association with dietary Mg^2+^ depletion in the present study may be the result of increased inflammatory and oxidative stress, as shown by NLRP3 inflammasome activation and IsoLG-adduct formation, respectively, of antigen presenting cells in the aorta. Future studies examining systemic vascular resistance and cardiac output may shed further insight on the mechanisms of how Mg^2+^ deficiency induces increases in blood pressure.

Magnesium plays a role in multiple cellular functions, and its deficiency has a significant impact on inflammatory processes. Limiting dietary magnesium by just 10% induces leukocyte migration, tissue infiltration, and activation to release inflammatory cytokines and free radicals (Classen et al., 1993; Malpuech-Brugere et al., 1999; Zimowska et al., 2002). During transendothelial migration, infiltrating monocytes differentiate into DCs (Randolph et al., 1998). Indeed, we found kidney DCs but not monocytes were activated by dietary Mg^2+^ depletion. Both myeloid-derived NLRP3 inflammasome and IsoLGs contribute to the pathogenesis of hypertension (Pitzer et al., 2020; Elijovich et al., 2021). This study represents the first demonstration that dietary magnesium depletion stimulates the NLRP3 inflammasome and production of IsoLGs. The observation that decreased extracellular Mg^2+^ concentration activates IL-1β production in cell culture demonstrates a direct stimulatory effect of Mg^2+^-depletion on dendritic cell activity. NF-κB is a transcription factor responsible for upregulation of NLRP3 inflammasome components. A study using neonatal monocytes found that magnesium sulfate supplementation inhibits NF-κB activity (Sugimoto et al., 2012). However, whether NF-κB plays a role and the precise mechanisms by which decreased extracellular Mg^2+^ activates these cells will require further study.

This study represents the first study showing that dietary Mg^2+^ depletion activates the NLRP3 inflammasome and stimulates production of IsoLGs, both of which promote hypertension. Given the prevalence of hypertension and of dietary Mg^2+^ depletion in human populations, the findings suggest that dietary Mg^2+^ consumption may represent an important, modifiable risk factor for hypertension and cardiovascular disease.

## Data Availability

The original contributions presented in the study are included in the article/Supplementary Material, further inquiries can be directed to the corresponding authors.

## Supplementary material

SUPPLEMENTARY FIGURE S1 Flow cytometry representative scatter plots showing the gating strategy to investigate NLRP3, IL-1β, and IsoLG-adducts in DCs and monocytes. SSCa, side scatter area.
