# Supplementary figures and images for: The role of dietary magnesium deficiency in inflammatory hypertension
Source: Front Physiol. 2023 May 24;14:1167904. doi: 10.3389/fphys.2023.1167904 (PMC10244581; doi:10.3389/fphys.2023.1167904)

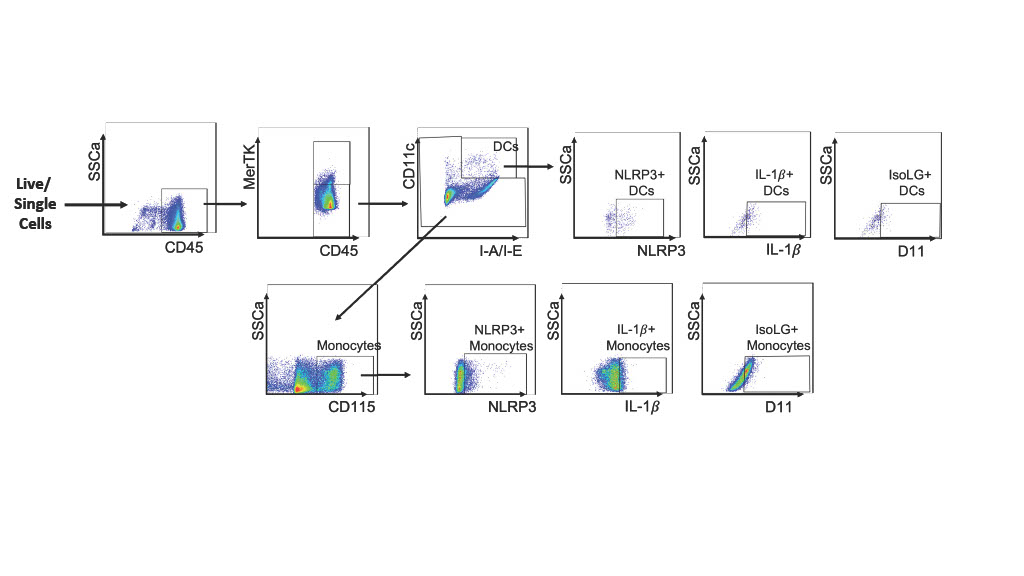

Supplement: Supplementary file 1 [file Image1.JPEG]
